# The Pathology, Diagnosis and Treatment of the Diseases of Women

**Published:** 1888-08

**Authors:** 


THE PATHOLOGY, DIAGNOSIS AND TREATMENT OF THE DISEASES OF WOMEN. By Graily Hewitt, M. D., Lond., F. R. C. P., Professor of Midwifery, etc. A New American, from the Fourth Revised and Enlarged London Edition. Edited with Notes and Additions by H. Marion Sims, M. D., New York. In three volumes. New York: E. B. Treat. 1887.

This work is a literal reprint of the one issued by Bermingham & Co. in 1883, and appears to have been printed from the same plates. The only addition is an appendix of four pages, containing descriptions of Sims' Retroversion Pessary and Sims' Abdominal Drainage Tube. No allusion is made to the many marked advances in gynæcology during the period which has elapsed since the appearance of the former edition. In short, it is five years behind the times. It fails, therefore, to represent the present status of the specialty of which it treats, and is unreliable as a guide to practice.

